# Investigation of Chloride Salt Erosion on Asphalt Binders and Mixtures: Performance Evaluation and Correlation Analysis

**DOI:** 10.3390/ma18010156

**Published:** 2025-01-02

**Authors:** Xin Qiu, Jianghui Deng, Qinghong Fu, Yunxi Lou, Yingci Ye, Dingchuan Zhang

**Affiliations:** College of Engineering, Zhejiang Normal University, Jinhua 321004, China; xqiu@zjnu.cn (X.Q.); dengjianghui@zjnu.edu.cn (J.D.); theresalou@163.com (Y.L.); yeyingci@zjnu.edu.cn (Y.Y.); dingchuanzhang@zjnu.edu.cn (D.Z.)

**Keywords:** chloride salt erosion, asphalt binder, asphalt mixture, performance evaluation, water stability, grey correlation analysis

## Abstract

Asphalt pavement, widely utilized in transportation infrastructure due to its favourable properties, faces significant degradation from chloride salt erosion in coastal areas and winter deicing regions. In this study, two commonly used asphalt binders, 70# base asphalt and SBS (Styrene–Butadiene–Styrene)-modified asphalt, were utilized to study the chloride salt erosion effect on asphalt pavement by immersing materials in laboratory-prepared chloride salt solutions. The conventional properties and adhesion of asphalt were assessed using penetration, softening point, ductility, and pull-off tests, while Fourier transform infrared spectroscopy (FTIR) elucidated the erosion mechanism. The Marshall stability test, freeze–thaw splitting test, and Cantabro test were applied to study the effects of chloride exposure on the strength, water stability, and structural integrity of the asphalt mixture. Finally, the grey correlation analysis was employed to assess the impact of chloride salt erosion on the performance of asphalt binders and mixtures. The findings highlight that chloride salt erosion reduces penetration and ductility in both types of asphalt binders, raises the softening point, and weakens asphalt–aggregate adhesion, confirmed as a primarily physical effect by FTIR analysis. Asphalt mixtures showed decreased strength and water stability, intensifying these impacts at higher chloride concentrations and longer erosion duration. SBS-modified asphalt binders and mixtures exhibited greater resistance to chloride salt erosion, particularly in adhesion, as demonstrated by the Cantabro and pull-out tests. Grey relational analysis revealed that erosion duration is the most influential factor, with *TSR* and softening point emerging as the most responsive indicators of chloride-induced changes. These findings offer critical insights for practice, providing evidence-based guidance for designing and constructing asphalt pavements in environments with high chloride levels.

## 1. Introduction

Asphalt binders and mixtures serve as critical components for pavement engineering and construction, offering essential properties such as durability, flexibility, and load-bearing capacity that ensure the reliability and longevity of pavement structures [1,2,3]. However, prolonged exposure to complex environments is a key factor leading to the durability loss and structural degradation of asphalt pavement [4,5]. Among them, ageing and water damage are the most prominent issues. Ageing causes the asphalt binder to undergo oxidation and polymerization, which increases its viscosity while reducing its flexibility, which negatively affects its rheological properties [6,7]. Water damage, often accompanied by chloride salt solutes [8], creates a chloride-rich environment that accelerates the degradation of the performance and structural integrity of asphalt binders and mixtures [9,10]. In northern China, where pavements often experience snow and ice during winter, chloride-based deicers are commonly applied to quickly eliminate snow and ice on roads, alleviating traffic disruptions [11]. Under freeze–thaw cycles, these chloride deicers gradually infiltrate the asphalt pavement structure [12]. The migration and accumulation of chloride ions lead to asphalt film peeling and a reduction in asphalt–aggregate adhesion [13,14]. Sea fog is a common phenomenon in the coastal areas of eastern China, with sea salt components dispersing in ion form carried by water vapour. When the water vapour condenses on the pavement surface, sea salt penetrates the bond between asphalt and aggregate, weakening the mechanical properties of the mixture [15].

The impact of chloride salts on asphalt pavement performance is a significant concern, particularly in cold or coastal regions. Studying the effects of chloride salt erosion on asphalt materials is crucial for understanding performance deterioration under various environmental conditions and traffic loads [16]. Jiang et al. [17] investigated asphalt–aggregate systems under freeze–thaw cycles in salt solutions, finding significant degradation in adhesion and the occurrence of irreversible fatigue after multiple cycles. Zhang et al. [18] conducted a performance evaluation of four different asphalt mixtures after immersion in a 10% chloride salt solution. Their findings revealed that chloride salt exposure caused surface damage, leading to a reduction in stability and stiffness, with polymer-modified asphalts exhibiting less sensitivity to chloride salts. Similarly, Juli-Gándara et al. [19] assessed the interaction between chloride salts and hot mix asphalt (HMA) mixtures as well as porous asphalt mixtures. They observed that while HMA showed minimal effects, chloride salt significantly impaired the internal friction of porous mixtures, resulting in decreased performance. Further research by Jiang et al. [20] examined the impact of dry–wet and freeze–thaw conditions on asphalt mixtures exposed to chloride salts. Their results showed that chloride salt crystals altered the roughness of aggregates, reducing the anti-sliding properties of the asphalt mixture but enhancing its tensile strength.

Understanding the interactions between chloride salts and the asphalt binder is essential for a comprehensive evaluation of chloride salt erosion mechanisms and their effects on both individual components and the final pavement structure. Peng et al. [20] found that the presence of chloride salt erosion increased the resistance ability of SBS-modified asphalt to high-temperature rutting and creep deformation. In the interaction between chloride salts and pure asphalt and aged asphalt, the effect on pure asphalt is more serious [21], and most of this effect is the penetration and adsorption of ions [22]. The mechanisms behind the chloride salt erosion of asphalt materials have been explored by several researchers. Jiang et al. [23] mentioned that the main mechanisms by which chloride salts erode asphalt binders include asphalt softening and viscosity reduction. Cai et al. [24] employed molecular dynamics simulations to study how asphalt ages when exposed to chloride salts, concluding that they increase the fraction-free volume (FFV) of asphalt, which heightens its moisture sensitivity to loss. Long et al. [25], using atomic force microscopy (AFM) to explore the micro-mechanisms of erosion by chloride salts, found that chloride exposure results in an amorphous film forming on the asphalt surface.

The impacts of chloride salt attack on asphalt pavement are multifaceted; substantial research has examined various environmental impacts on asphalt binders or mixtures and has identified pathways through which chloride salts deteriorate their properties, primarily focusing on changes in mechanical properties such as adhesion, tensile strength, and water resistance [26,27,28,29]. However, gaps remain in the understanding of the combined effects of multiple factors, such as salt concentration, exposure duration, and asphalt types, on the diverse performance of asphalt binders and mixtures. This highlights a critical knowledge gap, especially regarding predictive tools that could aid in assessing asphalt durability under chloride exposure. To address these issues, grey relational analysis was employed to perform a sensitivity analysis, which helped to determine the relative influence of various factors on the performance of asphalt binders and mixtures under chloride salt erosion conditions. It is well suited for evaluating complex interactions between multiple degradation factors due to its effectiveness in analyzing systems with incomplete or uncertain information [30,31].

This study aims to address these gaps by investigating the performance evolution of 70# base asphalt and SBS-modified asphalt binders and mixtures after immersion in chloride salt solutions at varying concentrations and durations. First, laboratory simulation tests were conducted by immersing asphalt binders and mixtures in chloride salt solutions to replicate real-world erosion conditions. Key properties, such as penetration, softening point, ductility, and adhesion, were assessed for asphalt binders, while FTIR was used to explore the underlying erosion mechanism. Marshall stability tests, freeze–thaw splitting strength tests, and Cantabro tests were conducted to evaluate the performance deterioration of the asphalt mixture by capturing changes in mechanical strength, water stability, and structural durability. From the measured values, this study establishes the relationship between chloride concentration, immersion duration, and the degradation of asphalt performance, offering a detailed understanding of chloride salt erosion effects. By quantifying the relationship between different chloride concentrations, erosion durations, and the evolution of the performance of asphalt binders and mixtures, this study seeks to offer a thorough analysis of the impacts of chloride salt erosion and important practical guidance for selecting and designing more durable asphalt materials for use in deicing salt environments.

## 2. Materials and Methods

### 2.1. Materials

#### 2.1.1. Asphalt

Two asphalt materials, including 70# base asphalt and SBS-modified asphalt, both sourced from Jinhua Xunda Construction Co, Ltd. (Jinhua, China), were used. Their basic properties are presented in Table 1.

#### 2.1.2. Aggregates

The asphalt mixtures incorporated coarse and fine limestone aggregates, along with limestone mineral powder, all supplied by Jinhua Xunda Construction Co., Ltd. The technical specifications for the three aggregates all conformed to the Technical Specifications for Construction of Highway Asphalt Pavements (JTG F40-2004) [33].

#### 2.1.3. Solute and Solvent

Sodium chloride (NaCl) and tap water were selected as the solute and solvent of the chloride salt solution in this research experiment. The NaCl was procured from Sinopharm Chemical Reagent Co., Ltd. (Shanghai, China), with a purity of ≥99.5%. Before use, the NaCl was dried to eliminate residual moisture. The main elements and content of tap water are required to comply with local municipal water supply standards.

### 2.2. Asphalt Mixture Design

**Figure 1:**
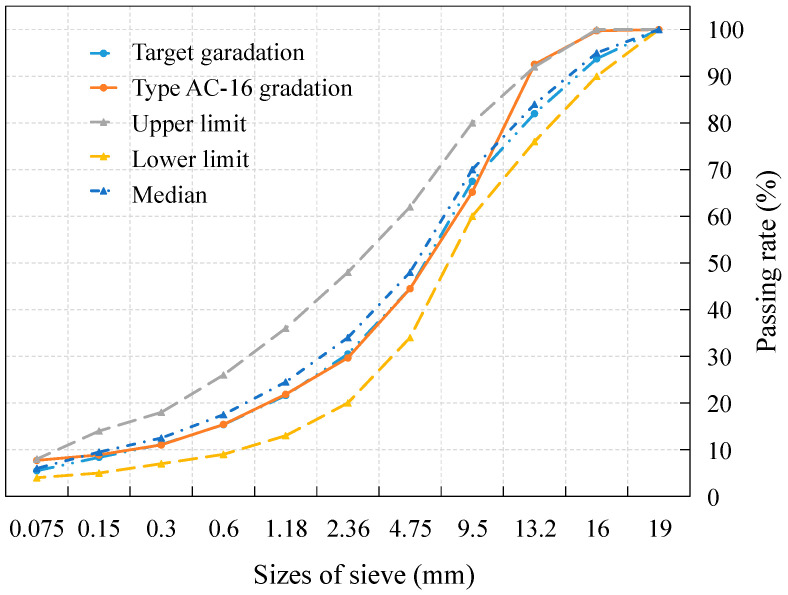
Design gradation curve of AC-16.

The Marshall design method was used for the AC-16 mixture design, with a target void ratio of 4%. The design gradation curve is shown in Figure 1. Marshall specimens were prepared with an asphalt–aggregate ratio ranging from 4.2% to 5.4%, at intervals of 0.3% [33]. Based on the evaluation of relevant indexes for each mixture, optimal asphalt–aggregate ratios were determined to be 4.8% for 70# base asphalt binder and 4.7% for SBS-modified asphalt binder.

### 2.3. Sample Preparation

**Figure 2:**
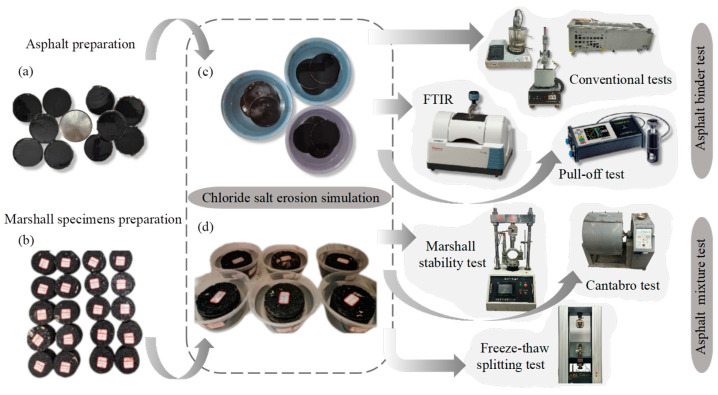
Sample preparation and test design of the asphalt and asphalt mixture (**a**) Asphalt binders for test; (**b**) Asphalt mixtures for test; (**c**) Erosion test of asphalt binders; (**d**) Erosion test of as-phalt mixtures.

Figure 2 illustrates the test design for preparing the asphalt and asphalt mixture samples.

#### 2.3.1. Chloride Salt Solution Preparation

Chloride salt solution with concentrations of 0%, 5%, 15%, and 25% were prepared by dissolving the appropriate mass of dried NaCl in tap water. The NaCl was weighed with an accuracy of 0.01 g, and stirring was performed at room temperature (25 °C) until the solution was completely dissolved. The 0% solution consisted of tap water without added NaCl and served as a control.

#### 2.3.2. Asphalt Preparation

To investigate chloride salt corrosion while eliminating the influence of asphalt film thickness, thin film specimens of 70# base asphalt binder and SBS-modified asphalt binder were prepared under actual construction conditions. Asphalt binder samples were poured into shallow trays 20 cm in diameter and 1 cm in height to ensure uniform film thickness. Each container was filled with 50 g of asphalt, which was heated to a pouring temperature of 130–140 °C for 70# base asphalt and 160–170 °C for SBS-modified asphalt to ensure fluidity and uniform distribution. After pouring, the specimens were cooled to room temperature in a controlled environment to stabilize the film structure before subsequent chloride salt erosion tests, as shown in Figure 2a.

#### 2.3.3. Marshall Specimen Preparation

Marshall tests were conducted to prepare asphalt mixture specimens according to Standard Test Methods of Bitumen and Bituminous Mixtures for Highway Engineering. The thickness of the cylindrical specimens of asphalt mixture specimens was 53.5 mm and the diameter was 101.6 mm; Figure 2b shows part of the specimens.

#### 2.3.4. Chloride Salt Erosion Simulation

The formed asphalt specimens and Marshall specimens were placed in chloride salt solutions of different concentrations, i.e., 0%,5%, 15%, and 25%, for durations of 1 day, 3 days, and 7 days (Figure 2c,d). The impact of chloride salt on the performance of asphalt and asphalt mixtures was examined by considering two factors: the concentration of chloride and the duration of erosion.

### 2.4. Test Methods

#### 2.4.1. Asphalt Tests

According to JTG E20 [32], the penetration, softening point, and ductility tests were used to evaluate the asphalt consistency, high-temperature performance, and low-temperature performance, respectively. The ductility test temperature was set at 15 °C to more accurately reflect the effects of chloride salt deicers and sea mist on the performance of SBS-modified asphalt in a low-temperature environment. Penetration and ductility tests were conducted with three replicates, while the softening point test was carried out with two replicates.

Asphalt binder–aggregate adhesion can be affected by chloride salt erosion conditions, resulting in reduced pavement performance. In this section, the PosiTesAT-A instrument (DeFelsko, West Chester, PA, USA) was used to measure the adhesion strength between the coating or material and the substrate. The number of replicates for the adhesion test was four.

FTIR is an advanced tool that can analyze the structure and composition of substances. In order to clearly observe the changes in asphalt microstructure before and after chloride salt erosion, samples were selected from uncorroded asphalt binders exposed to clean water and asphalt binders exposed to 5%, 15%, and 25% chloride salt solutions for 7 days. Testing was conducted using a Nicolet iS5 FTIR spectrometer equipped with a germanium crystal for attenuated total reflection (ATR). Thin films of asphalt binders were carefully cut from the specimen surface to ensure uniformity and placed directly on the germanium crystal for analysis, and data acquisition and analysis were performed using OMNIC software 9.2.86. Each sample was tested in triplicate.

#### 2.4.2. Asphalt Mixture Tests

Chloride salt erosion in the form of water damage occurs mainly in the coastal areas of eastern China, which are characterized by large amounts of chloride ions in sea fog and in areas characterized by the formation of chloride solutions after snowmelt due to the use of deicing agents. This phenomenon manifests itself on-site as water damage, which has a significant impact on the water stability of asphalt mixtures. In this section, the water stability of asphalt is investigated mainly through the Marshall test, the freeze–thaw splitting test, and the Cantabro test.

In the Marshall stability test, specimens exposed to various chloride concentrations and erosion durations were immersed in a 60 °C water bath for 30 min, after which, their stability was assessed.

The freeze–thaw splitting test involves more rigorous testing conditions and provides results that better simulate the actual performance of asphalt pavements. The splitting tensile strength ratio (*TSR*) is determined using Equations (1)–(3).

(1) $$RT_{1}=0.006287P_{T_{2}}÷h_{1}$$

(2) $$RT_{2}=0.006287P_{T_{2}}÷h_{2}$$

(3) $$TSR=\frac{RT_{2}}{RT_{1}}\times 100\%$$

where *TSR* is the freeze–thaw splitting residual strength ratio, *RT*_1_ is the splitting tensile strength of the first group of single specimens without freeze–thaw cycles, *RT*_2_ is the splitting tensile strength of the second group of single specimens subjected to freeze–thaw cycles, *h* is the specimen height, and *PT* is the maximum specimen load.

The Cantabro test serves as an important approach for assessing the durability or resistance to abrasion of an asphalt mixture. It involves subjecting a sample of asphalt mixture to mechanical agitation in a rotating drum, simulating the wear and tear on the pavement surface caused by traffic. The mass loss of the sample (Δ*S*) after a specified number of rotations indicates its abrasion resistance. A lower mass loss indicates better durability. The equation for calculating Δ*S* is as follows:

(4) $$\Delta S=\frac{m_{0}-m_{1}}{m_{0}}$$

where Δ*S* is the mass loss rate of the asphalt mixture, *m*_0_ is the mass of the asphalt mixture before testing, and *m*_1_ is the residual mass of the asphalt mixture following the test.

### 2.5. Grey Relation Analysis

The grey relational analysis method was employed to construct an analytical model of the effect of chloride concentration and erosion duration on the performance of asphalt and asphalt mixtures. Grey relational analysis is a technique that measures the degree of association between factors based on the similarity or dissimilarity of their development trends. This method is particularly effective in analyzing complex relationships between specified factors when dealing with insufficient or uncertain information [34]. The implementation of grey relational analysis consists of three main steps: first, determining the target series and comparison series; second, dimensioning these series; and finally, calculating the grey relational coefficients to derive the relational degree [35,36]. By comparing the magnitudes of the relational degrees, it is possible to rank the associations among the relevant factors.

Based on the test of this study, the penetration, ductility, softening point adhesion, Marshall stability, *TSR*, and Δ*S* were selected as target series, and the chloride solution concentration and erosion time were selected as comparison series. The two are expressed by Equations (5) and (6), respectively.

(5) $$X_{0}(k)=\left\{x_{0}^{1},x_{0}^{2},\ldots ,x_{0}^{a}\right\},\;k=1,2,\ldots a$$

(6) $$X_{i}(k)=\left\{x_{i}^{1},x_{i}^{2},\ldots ,x_{i}^{a}\right\},\;k=1,2,\ldots b$$

Normalization is adopted as the dimensionless processing method in this paper; Equations (7) and (8) show the specific process.

(7) $$Y_{0}(k)=\frac{x_{0}^{a}(k)}{x_{1}^{a}(1)}$$

(8) $$Y_{i}(k)=\frac{x_{i}^{a}(k)}{x_{i}^{a}(1)}$$

where *Y*_0_(*k*) is the target series and *Y_i_*(*k*) is the comparison series post data pre-processing.

After nondimensionless processing of the data, the grey relational coefficient, which can assess the relationship between the target series and the comparison series, was calculated using Equations (9)–(12).

(9) $$\Delta_{0j}(k)=\left\|Y_{0}(k)-Y_{j}(k)\right\|$$

(10) $$\Delta_{\min}=\underset{\forall j\in i}{\min}\;\underset{\forall k}{\min}\left\|Y_{0}(k)-Y_{j}(k)\right\|$$

(11) $$\Delta_{\max}=\underset{\forall j\in i}{\max}\;\underset{\forall k}{\max}\left\|Y_{0}(k)-Y_{j}(k)\right\|$$

(12) $$\eta (x_{0}(k),x_{i}(k))=\left|\frac{\Delta_{\min}+\mu \Delta_{\max}}{\Delta_{oj}(k)+\mu \Delta_{\max}}\right|$$

where Δ_0_*_j_*(*k*) is the deviation series between target series *Y*_0_(*k*) and comparison series *Y*_i_(*k*) at moment k. *μ* is the identification coefficient, usually assigned a value of 0.5. *ƞ*(*x*_0_(*k*), *x_i_*(*k*)) is the grey relational coefficient.

Then, the grey relational degree can be calculated by Equation (13).

(13) $$\lambda_{0i}=\frac{1}{n}\sum_{k-1}^{n}\eta (x_{0}(k),x_{i}(k))$$

## 3. Test Results and Analysis

### 3.1. Conventional Performance

**Figure 3:**
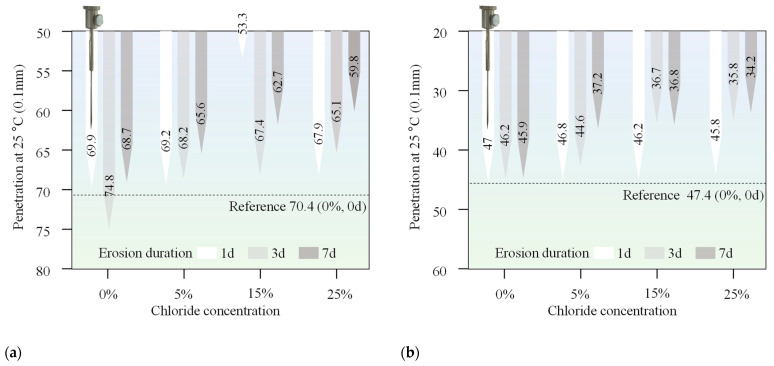
Penetration of (**a**) 70# base asphalt binders and (**b**) SBS-modified asphalt binder after erosion in varying chloride concentration solutions for different durations.

**Figure 4:**
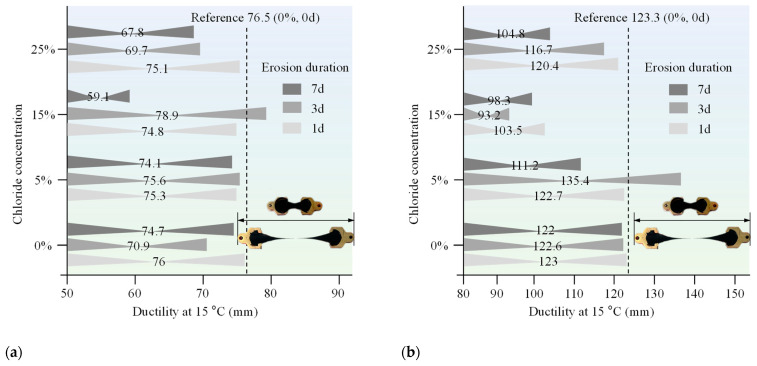
Ductility of (**a**) 70# base asphalt binder and (**b**) SBS-modified asphalt binder after erosion in varying chloride concentration solutions for different durations.

**Figure 5:**
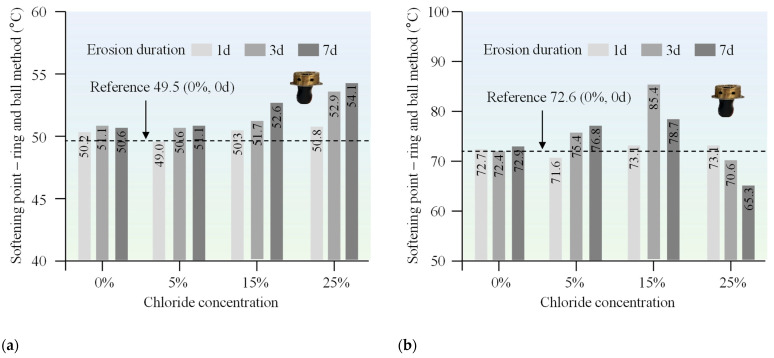
Softening point of (**a**) 70# base asphalt binder and (**b**) SBS-modified asphalt binder after erosion in varying chloride concentration solutions for different durations.

The penetration, ductility, and softening point of both 70# base asphalt and SBS-modified asphalt were measured after immersion in chloride salt solutions of varying concentrations and durations. The purpose of these tests was to assess how chloride environments affect the conventional properties of asphalt. The results are presented in Figure 3, Figure 4, and Figure 5, respectively.

The penetration of two asphalt binders exhibited a general decrease after immersion in chloride salt solutions, with a more significant decrease observed as the immersion duration increased from 1 day to 7 days. This trend was consistent for both types of asphalt binders, indicating that chloride salt erosion has a clear influence on the consistency characteristics of the materials. A decrease in penetration typically indicates an increase in the stiffness of the asphalt, which can negatively affect its low-temperature flexibility and cracking resistance. As the chloride salt solution penetrates the asphalt, it leads to the formation of salts within the asphalt matrix. This can promote the hardening of the asphalt, further increasing its stiffness. As the concentration of chloride salts increases, high concentrations of chloride ions can cause changes in the internal structure of the asphalt, strengthening the interaction between the chloride ions and the asphalt molecules and further increasing the hardness of asphalt binders. Additionally, prolonged exposure to chloride salts accelerates the process, as evidenced by the greater reduction in penetration with longer erosion durations.

Chloride salt erosion generally reduces the ductility of asphalt; chloride salts accelerate the hardening of asphalt by promoting the formation of crystalline salts within the asphalt. This crystallization process limits the asphalt’s ability to deform, thereby reducing its ductility.

As shown in Figure 5, chloride salt exposure increased the softening point of asphalt, reflecting an improvement in its high-temperature performance. The asphalt samples soaked in higher concentrations of chloride salts showed higher softening points, which is similar to the mechanism of chloride salts on ductility. The higher concentration of chloride ions enhances the interaction between asphalt molecules, such as van der Waals forces or hydrogen bonds, and this increased force allows the asphalt to begin to soften at higher temperatures. Interestingly, a comparison of the softening point between 70# base asphalt and SBS-modified asphalt after exposure to the chloride salt was made. The bar chart illustrates the differences in softening point between base asphalt and SBS-modified asphalt at different chloride salt concentrations. SBS-modified asphalt showed a smaller increase in softening point compared to 70# base asphalt, also suggesting that the polymer modification helps to resist salt-induced hardening.

### 3.2. Adhesion

**Figure 6:**
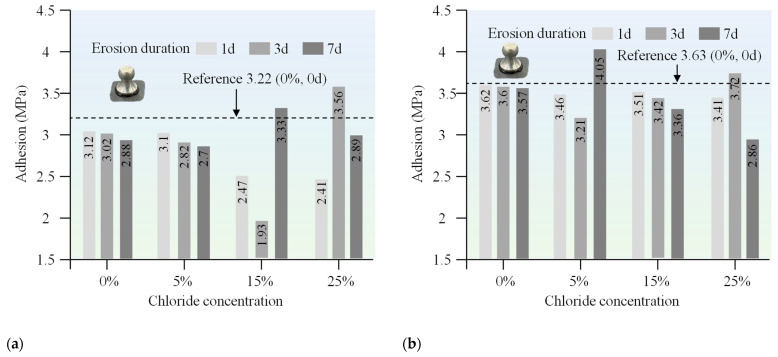
Adhesion of (**a**) 70# base asphalt binder and (**b**) SBS-modified asphalt binder after erosion in varying chloride concentration solutions for different durations.

The adhesive strength of 70# base asphalt and SBS-modified asphalt generally decreased as the chloride concentration and erosion durations increased, as shown in Figure 6. Throughout the test period, the adhesive strength of SBS-modified asphalt remained consistently higher than that of the 70# base asphalt. Moreover, the rate of change in bond strength was more gradual for SBS-modified asphalt, suggesting that it has superior stability in adhesive performance under chloride salt erosion compared to the 70# base asphalt.

When exposed to elevated chloride concentrations (i.e., 15% and 25%), the adhesive strength of both 70# base asphalt and SBS-modified asphalt binders showed potential increases over time. This phenomenon may be due to interactions between certain asphalt components and chloride ions during the later stages of erosion, which may have strengthened the adhesion between the asphalt and the aggregate. However, the mechanism responsible for this adhesion improvement remains to be further explored.

### 3.3. FTIR

**Figure 7:**
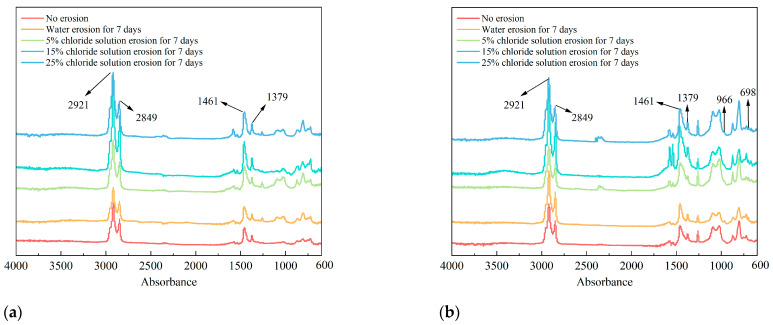
The FTIR test result of (**a**) 70# base asphalt binder and (**b**) SBS-modified asphalt binder after erosion in varying chloride concentration solutions for 7 days.

The infrared spectra of asphalt binders under different chloride salt erosion conditions are shown in Figure 7. The absorption peaks near 2921 cm^−1^ and 2849 cm^−1^ correspond to the vibration of saturated bonds. The peaks at 1600–1550 cm^−1^ correspond to the stretching vibrations of C=C bonds, characteristic of aromatic rings in asphalt. For the SBS-modified asphalt binder, the intensity of these peaks is generally higher than that of the 70# base asphalt binder due to the presence of the styrene segments in SBS, which are rich in aromatic structures. C-H stretching vibrations of methyl and methylene were observed, and the C-H vibrations occur at 1460 cm^−1^ and 1375 cm^−1^, which are the in-plane bending vibrations of methyl and methylene C-H. The peaks at 1100–1050 cm^−1^ are associated with the stretching vibrations of C–O bonds, indicating the presence of polar functional groups such as esters or ethers. In SBS-modified asphalt, these peaks may also reflect contributions from the butadiene segments. The absorption peaks near 966 cm^−1^ and 698 cm^−1^ in the Fourier infrared spectrum of SBS correspond to the out-of-plane bending vibrations of olefinic C-H, indicating the presence of SBS modifier in the asphalt binder.

A comparison of the characteristic peaks before and after erosion reveals that the absorption peak positions of both types of asphalt remained unchanged after exposure to varying chloride concentrations and erosion durations. However, the intensity of these absorption peaks exhibited noticeable changes. This indicates that the erosion effect of chloride salts on asphalt primarily involves the breaking and reorganization of chemical bonds within the asphalt, without the formation of new substances. In other words, the impact of chloride salts on asphalt is a physical process rather than a chemical reaction. Furthermore, as the chloride concentration increased and the erosion time extended, this physical effect intensified, leading to significant changes in the intensity of the absorption peaks. To comprehensively confirm this conclusion, future experiments will consider using Differential Scanning Calorimetry (DSC) and Wide-Angle X-ray Diffraction (WAXD). These techniques will provide valuable insights into both thermodynamic parameters and microstructural analysis, providing further support for the physical nature of the interaction between chloride salts and asphalt [37].

### 3.4. Marshall Stability

**Figure 8:**
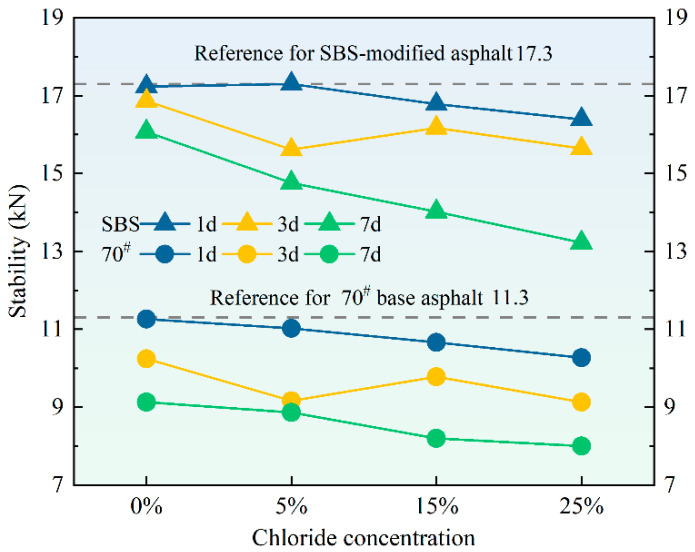
The Marshall stability of 70# base asphalt mixtures and SBS-modified asphalt mixtures after erosion in varying chloride concentration solutions for different durations.

Figure 8 shows the stability of asphalt mixtures under varying chloride salt concentrations and erosion durations. The stability of asphalt mixtures is an important measure of their ability to resist deformation under traffic loading [38]. The analysis of the experimental data reveals that the stability of the asphalt mixture decreased after immersion in chloride salt solutions, and this decline was more significant with higher concentrations and longer erosion durations. The trends observed for both types of asphalt were similar, indicating that chloride salt exposure generally affects the stability of both 70# base and SBS-modified asphalt mixtures, although the SBS-modified asphalt mixtures exhibited slightly higher stability. A decrease in stability typically suggests a weakening of the strength and permanent deformation of asphalt mixtures, which may undermine the functionality of road surfaces.

The observed reduction in stability following chloride salt exposure can be attributed to the chloride salts penetrating into the asphalt matrix, resulting in a disturbance of the molecular structure of the asphalt binders. This disturbance is likely to reduce the bond between asphalt and aggregates, resulting in reduced mixture integrity. As the concentration and immersion time of the chloride salt increased, the salt ions more effectively interacted with the asphalt, causing further degradation, consistent with the observed trend of decreasing stability.

### 3.5. Freeze–Thaw Splitting Test

**Figure 9:**
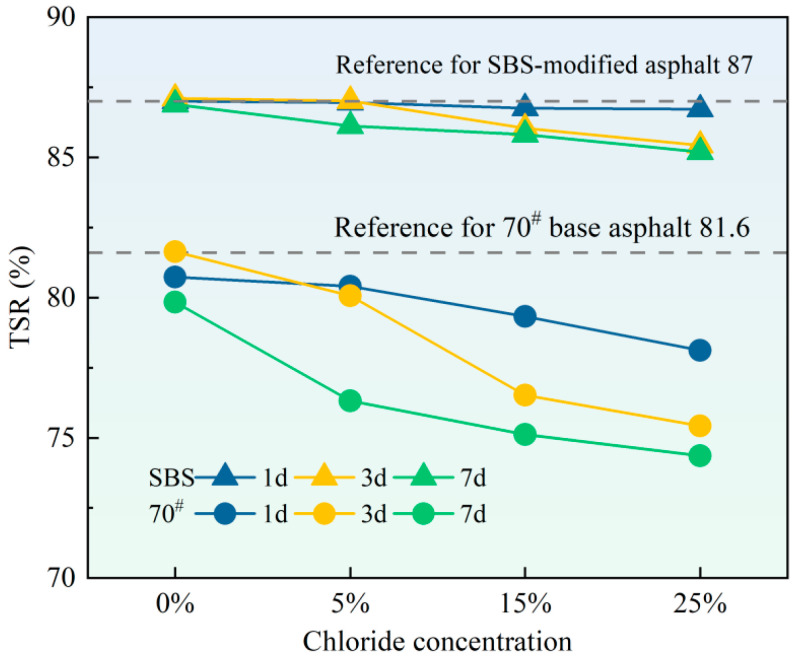
*TSR* of 70# base asphalt mixtures and SBS-modified asphalt mixtures after erosion in varying chloride concentration solutions for different durations.

Figure 9 shows the freeze–thaw splitting result; the presence of chloride significantly reduced the freeze–thaw splitting strength ratio of both 70# base asphalt and SBS-modified asphalt mixtures compared to water erosion. This deterioration significantly reduced their crack resistance under low-temperature conditions. Specifically, as the chloride concentration and erosion duration increased, the splitting strength ratio of the asphalt mixtures exhibited a downward trend, indicating an overall degradation in splitting strength, with the degree of deterioration becoming more pronounced with higher concentrations and longer exposure times. Comparatively, the splitting strength ratios of the two asphalt mixtures demonstrate that SBS-modified asphalt mixtures primarily maintained a splitting strength ratio between 80% and 90%. In contrast, the 70# base asphalt mixtures showed a ratio between 70% and 80%, indicating a superior resistance to salt erosion in the SBS-modified asphalt mixtures.

### 3.6. Cantabro Test

**Figure 10:**
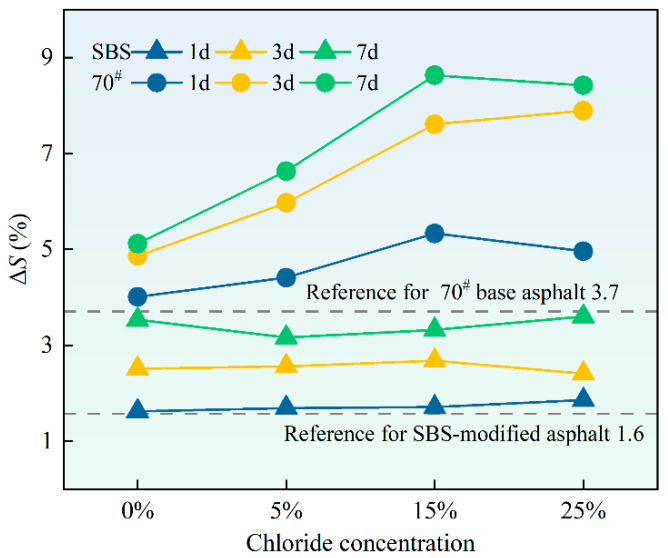
Δ*S* of 70# base asphalt mixtures and SBS-modified asphalt mixtures after erosion in varying chloride concentration solutions for different durations.

The data presented in Figure 10 illustrate a clear trend where the loss rate correlates with chloride salt concentrations and erosion duration. In the low-concentration chloride solution, the loss rate of asphalt mixtures increased rapidly at the beginning due to the physical erosion effect of the chloride solution. Chloride solution infiltrated the fractures within the asphalt mixture, causing crystallization erosion, which is an important cause of performance degradation. Over time, the erosion of the chloride solution would gradually reach saturation, so the flying loss rate would tend to be flat. This shows that in a high-concentration chloride environment, the performance degradation of asphalt mixtures will experience a process of rapid decline and then stabilization.

Furthermore, at equivalent chloride concentrations and exposure times, the SBS-modified asphalt mixtures consistently exhibited lower spalling losses than the 70# base asphalt mixtures, demonstrating its superior resistance to spalling. This may be related to the structure of SBS-modified asphalt, which enhances elasticity and resistance to ageing by incorporating an SBS modifier. However, base asphalt has poorer erosion resistance and is therefore more susceptible to flying losses under chloride salt erosion.

### 3.7. Correlation Analysis of Asphalt and Asphalt Mixture Performance

**Figure 11:**
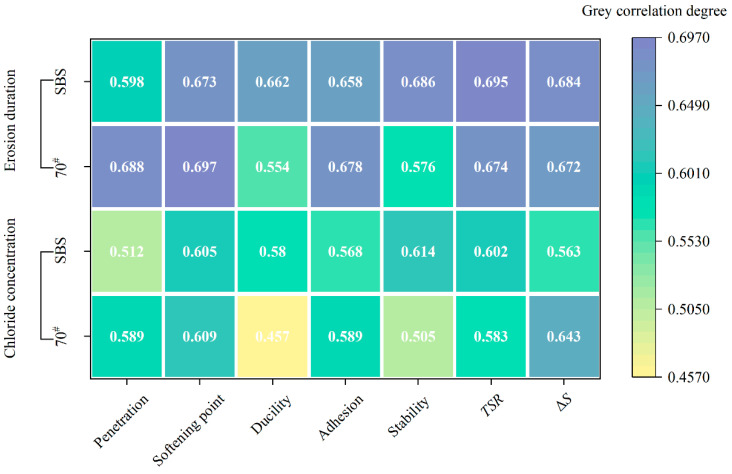
Grey correlation degree of different asphalt performance indexes.

The grey correlations between the chloride concentration, erosion duration, and the characteristic of two asphalt binders and mixtures are presented in Figure 11.

The grey relation analysis revealed that erosion duration had a higher degree of grey relation with the erosion performance of both the asphalt and its mixtures than chloride salt concentration. This suggests that prolonged exposure to chloride salt solutions has a greater impact on the deterioration of asphalt and its mixtures than the concentration of chloride salt itself. Longer immersion times allow more time for the chloride ions to penetrate the asphalt matrix, leading to greater degradation of the material properties. This finding is consistent with previous studies that indicate time-dependent processes, such as absorption and diffusion, are crucial in determining the long-term performance of asphalt under environmental stresses [39].

The results also indicated that the performance of the asphalt mixtures was more strongly influenced by chloride salt exposure than the asphalt binder alone. This is probably due to the complex interactions between the aggregate and asphalt in the mixture. The presence of aggregates provides additional sites for chloride salt absorption, while the properties of the asphalt, including adhesion and cohesion, are further weakened by the chloride ions. As the mixture is more heterogeneous than pure asphalt, its performance is more sensitive to external conditions, such as chloride salt exposure, leading to more pronounced changes in stability and strength.

Based on Figure 11, there was a high degree of correlation between the softening point and *TSR*. Interestingly, the softening point of both 70# base and SBS-modified asphalt increased after chloride salt erosion. This phenomenon can be explained by the interaction of the chloride ions with the asphalt binder, which may have caused the binder to undergo a plasticization process, leading to a higher softening point. As chloride salts infiltrate into asphalt, the molecular structure of the binder can be altered, potentially making the asphalt more resistant to softening at high temperatures. Where the binder softens and loses its structural integrity, this increase in softening point indicates that the chloride ions may act as a stabilizing agent in certain conditions, possibly due to the formation of ionic bonds within the asphalt matrix that mitigate excessive softening [5]. This observation highlights a unique response of asphalt materials to chloride salt exposure, differing from typical high-temperature performance degradation mechanisms. The decrease in *TSR* observed in this study is consistent with the understanding that chloride salts exacerbate the moisture sensitivity of asphalt mixtures, especially under freeze–thaw conditions. The presence of salt accelerates the absorption of water into the binder, which can cause significant damage to the asphalt microstructure when subjected to freezing and thawing. The cyclic freezing and thawing, combined with the corrosive effects of chloride salts, could lead to the expansion and contraction of water within the pores of the asphalt mixture, further weakening the material.

Future research and pavement design must focus on mitigating the adverse effects of chloride salts on water stability to extend the lifespan of pavements in areas where salt deicers are used or near the sea. Engineers need to account for both the duration of chloride exposure and overall durability of the pavement under freeze–thaw conditions. While chloride salt may improve high-temperature performance, prioritizing water stability through targeted material modifications will ensure the extended service life of asphalt pavements in chloride-rich environments.

## 4. Conclusions

This study investigates the performance evolution of 70# base and SBS-modified asphalt binders and mixtures after exposure to chloride salt solutions at various concentrations and durations. The results indicate several key findings, which are as follows:

(1) Chloride salt erosion significantly reduced the penetration and ductility of two asphalt materials, while increasing the softening point and weakening the adhesion between the asphalt binder and aggregates. FTIR confirmed that chloride salt erosion was a primarily physical process.

(2) For the asphalt mixture, both the strength and water stability were decreased by chloride salt erosion, and these effects worsened with increasing chloride concentration and erosion duration.

(3) SBS-modified asphalt showed superior resistance to chloride salt erosion compared to 70# base asphalt, with less degradation in key performance indicators. In particular, chloride salt had minimal effect on the adhesion of SBS in the Cantabro test, whereas it significantly weakened the adhesion between 70# base asphalt and the aggregate, consistent with the pull-out test results.

(4) Grey relation analysis indicated that the duration of chloride salt erosion had the most significant impact on the performance of asphalt and asphalt mixtures. Notably, *TSR* and softening point were identified as the most sensitive indicators of chloride-induced evolution.

This study provides valuable insights into the chloride salt erosion resistance of different asphalt types. SBS-modified asphalt, due to its polymer-enhanced structure, exhibited better resistance to chloride-induced degradation, particularly in terms of adhesion. This work contributes to the existing literature by systematically evaluating the effects of chloride salt exposure on asphalt and asphalt mixtures and identifying key performance indicators sensitive to chloride salt erosion. The findings emphasize the need for incorporating SBS modification in regions exposed to deicing salts and provide a reference for future pavement design and maintenance strategies.

## Figures and Tables

**Table 1:** Main properties of the asphalt binders.

Items	Unit	Standard [32]	70#	SBS
Penetration at 25 °C	0.1 mm	JTG E20 T0604	70.4	47.4
Softening point	°C	JTG E20 T0606	49.45	72.6
Ductility at 15 °C	cm	JTG E20 T0605	123.25	76.5
Density	g/cm^3^	JTG E20 T0603	1.024	1.034

## Data Availability

Data will be made available on request.
